# The Relationship Between Bullying and Organized Sports Participation Among Sexual and Gender Diverse Youth: A Narrative Review and Directions for Inclusive Programs

**DOI:** 10.3390/ijerph23070903

**Published:** 2026-07-15

**Authors:** Linh-Nhu Hoang, Stefan Nikolic, Mahesh Shrestha, Dilip R. Patel

**Affiliations:** 1 Department of Psychology, Western Michigan University, Kalamazoo, MI 49008, USA; nhu.hoang@wmich.edu; 2 Department of Pediatric and Adolescent Medicine, Western Michigan University Homer Stryker M.D. School of Medicine, Kalamazoo, MI 49008, USA; stefan.nikolic@wmed.edu (S.N.); mahesh.shrestha@wmed.edu (M.S.)

**Keywords:** bullying, mental health, sports participation, youth, sexual and gender diverse

## Abstract

**Highlights:**

**Public health relevance—How does this work relate to a public health issue?**

- Bullying, with its longstanding concern worldwide, has been defined and studied for several decades as a public health concern for youth.
- The effects of bullying can lead to long-lasting negative consequences for one’s mental health and has been one of the main priorities of public health guidelines.

**Public health significance—Why is this work of significance to public health?**

- Increasing participation in sports and physical activity, particularly during youth development, is a public health priority in many countries.
- The connections between organized sport involvement and mental health among youth are heavily underexplored as national and multi-state estimates of organized team sport participation can be difficult to obtain and interpret.

**Public health implications—What are the key implications or messages for practitioners, policy makers and/or researchers in public health?**

- Understanding the current and negative experiences with physical activity among students with negative experiences will be useful for practitioners and administrators in influencing and creating infrastructures and programming within the schools toward safe, supportive, and inclusive environments for physical activity.
- While there may be several attempts to engage relevant stakeholders—teachers, coaches, and parents—in enhancing anti-bullying strategies, there is a lack of evidence-based recommendations, particularly for sexual and gender diverse (SGD) youth. More research is required to understand the efficacy of such training programs and interventions to empower SGD youth athletes in organized sports.

**Abstract:**

Participation in organized sports has been shown to enhance youths’ development, improve mental health symptoms, and maintain overall well-being. Research on the outcomes of bullying in the youth population shows the opposite. Youth from marginalized communities in particular—specifically sexual and gender minority or lesbian, gay, bisexual, transgender, queer, intersex, asexual (LGBTQIA+) youth—are at an increased risk of being victims of bullying than their counterparts. However, there is a gap in the literature on the relationship between the three variables. Thus, this review explored the relationship between bullying among sexual and gender minority youth and its impact on the level of engagement in organized sports participation. Psychosocial risk factors and responses are identified, and suggestions for sports programs are proposed. A total of 16 articles, categorized by sexual and gender identity groups to further highlight the current literature on specific groups, were included in the final synthesis of this review. The findings revealed a range in the experiences of bullying and levels of sports participation among LGBTQIA+ youth, highlighting individual differences and impacts of various factors. The findings also emphasized the importance of involvement from schools, administrators, and policymakers in creating a safer, inclusive environment for LGBTQIA+ youth to increase engagement in sports, ultimately improving overall well-being and development.

## 1. Introduction

Bullying is notably prevalent and unfortunately common among children and adolescents, especially in the context of the physical education (PE) environment [1]. Physical activity can serve as a protective factor that promotes health development and well-being among children and adolescents, as it is important for preventing chronic disease and improving physical development and cognitive functioning. Organized sports provide such opportunities for participation in physical activity, leading to several health benefits and positive outcomes [2]. Thus, physical education is the environment that allows opportunities for organized (team) sports, which includes the act of physical activity. Physical activity is associated with many physical and mental health benefits, including reduced risks of cancer, diabetes, and early mortality, as well as reduced risks of depression and anxiety [3]. Additionally, youth who participate in sports often report higher levels of self-esteem, self-confidence, and resilience, as well as improved social skills [2,4]. For that reason, increasing participation in sports and physical activity, particularly during youth development, is a public health priority in many countries. However, the literature on the association between physical activity and negative safety and violent experiences among youth, particularly bullying, has not been studied extensively with nationally representative data [5]. The following subsections provide background on the topics that helps us understand the aim of this narrative review on the relationship between bullying and sports participation among LGBTQIA+ youth.

### 1.1. Bullying and Mental Health

Bullying, with its longstanding concern worldwide, has been defined and studied for several decades as a public health concern for youth. Bullying can be broadly conceptualized as a series of deliberate and negative behaviors, including verbal, physical, or social behaviors, from an individual who holds power and privilege over another individual or group of individuals [6,7]. It can take form in two ways: the traditional face-to-face form and the electronic form, often understood as cyberbullying [1]. Though the majority of school bullying is performed by the student’s peer or peers, bullying can also be performed by the student’s teachers, paraeducators, or school personnel [1]. The negative behavior can then result in physical, mental, or emotional harm to victims of bullying. Such harm can include, but is not limited to, low self-esteem, feelings of loneliness, and increased suicidal ideation among youth [6].

Bullying appears to influence mental health outcomes, particularly depressive symptoms in children and adolescents. School bullying, in particular, has been shown to have profound and lasting effects on the health and well-being of those impacted, and it is closely associated with symptoms related to depression, anxiety, insomnia, and suicidal ideation [8]. Bullying often peaks between the ages of 11 and 13 years, which is often the transition from primary to secondary school. Adolescents, by the age of 18 years, are more than twice as likely to experience depression-related symptoms than those who were not bullied at the age of 13 years [7]. Bullying also has been found to increase self-harm thoughts and behaviors, as well as increasing the risk of suicidal ideation. The influence and effects of bullying experienced during childhood may even extend into adulthood [1]. This can lead to long-lasting negative consequences for one’s mental health, and understanding its impact has been one of the main priorities of public health guidelines on physical activity [5].

Several studies examined and emphasized the importance of intervention and peer-support programs in preventing bullying; however, much of the existing literature has relied on qualitative interviews and case studies [9]. While qualitative research is important in providing a depth of understanding, there is a lack of systematic methodology for assessing effective strategies.

### 1.2. Organized Sports Participation and Development

Organized sports, by definition, is the type of physical activity that is directed by an adult or youth leader, involving rules and formal competition [2]. The nature of competitive play, such as in a game of dodgeball, can lead to higher chances of eliciting aggressive behaviors, making vulnerable students susceptible targets for bullying [1]. The connections between organized sport involvement and mental health among youth are heavily underexplored, as national and multi-state estimates of organized team sport participation can be difficult to obtain and interpret [10]. It is imperative to consider various operational definitions of what an ‘organized sport’ entails from an international perspective. Additionally, this does not account for the fact that organized team sport participation among sexual and gender diverse youth is significantly lower than the general population. Several studies discussed in the paper include discussions of the relationship between bullying and sports participation among sexual and gender diverse youth from several international countries.

Despite the fact that only one in four youth meet global physical activity guidelines by the World Health Organization (WHO), physical activity has been proven to be an effective intervention in anti-bullying programs for various mental and physical health conditions among youth [11]. The youth population in this context includes individuals under the age of 26. Physical activity also demonstrates strong anti-depressive and anti-anxiety effects, improved efficacy, and enhanced mood regulation [10]. Interestingly, students who participate in sports have been found to show greater self-control, less aggressive attitudes, and more respectful behavior towards their peers and established rules [6]. This finding highlights the variability in sports participation outcome among youth, emphasizing why organized sports are a distinct context for further analysis. Epidemiological data in England has revealed that youth are less likely to meet physical activity guidelines according to socioeconomic factors, gender (e.g., girls and “other”), and ethnicity (e.g., Black, Asian, Latinx, and Other Non-White) [10]. Participation in sports can help mitigate the negative effects of bullying and serve as an effective strategy for promoting positive peer interactions and improving emotional regulation. Appropriate physical and cognitive development through a healthy lifestyle is crucial to overall well-being and development among the global youth population, and engagement in physical activity are among many healthy lifestyle habits. Sports practice helps promote a level of responsibility and pro-social attitudes [12].

### 1.3. Sexual and Gender Diverse Youth

In recent years, more children and adolescents are becoming comfortable with exploring and embracing their sexual and gender identity. Gender diverse youth—individuals who identify on the spectrum of gender identities—make up about 8.4% of the global child and adolescent population [3]. Per the Centers for Disease Control and Prevention (CDC) reports on sports participation rates, only about one-third of lesbian, bisexual, and gay (LBG) youth engaged in a team sport compared to their heterosexual counterparts [3]. This low percentage of sports participation for LBG youth suggests specific barriers exist in discouraging this population from joining an organized sports team. Sexual and gender orientation have been found to be risk factors for harassment in sports [2].

Transgender youth, in particular, make up about 2.7% of the global child and adolescent population [3]. Transgender individuals, otherwise referred to as ‘trans’ people, have a gender that differs from the sex assigned at birth, which includes trans men, trans women, and non-binary individuals. Trans youth are significantly underrepresented in sports and physical activities compared with their cisgender peers. Sports policies have historically been exclusionary of trans people, especially trans girls and trans women, which increasingly serves as a barrier to participation in sports and physical activities [3]. Since the first law that banned trans girls from participating in school sport teams, other states have increasingly passed similar laws [13].

## 2. Materials and Methods

The research team, including the authors and a medical librarian, conducted preliminary and focused database searches in PubMed and Scopus on 14 January 2026, focusing on the most recent literature to examine this research topic. Thus, the search was combined and filtered to include articles published within the last 10 years to capture relevant findings. The specific search strings are available in Appendix A, including the preliminary, focused, and combined searches. To be included in the final synthesis, articles needed to address the relationship between bullying and participation in organized sports for LGBTQIA+ youth or sexual and gender minority youth. Peer-reviewed articles, systematic reviews, narrative reviews, and meta-analyses were also included. Case reports, comments, or letters to the editor were excluded. Articles were also excluded if the participants were older than the World Health Organization (WHO)’s defined age for “youth” (i.e., 25+ years of age). However, the research team extended the cutoff to 26+ years of age, as this is often extended depending on the context. Additionally, articles that lacked LGBTQIA+ and sports participation presence, as well as articles that were incomplete or unable to be retrieved, were excluded. Titles, abstracts, and full-text articles were screened by the first and second authors of this manuscript, and the authors utilized the inclusion and exclusion criteria to select finalized articles to be included in this narrative review. Further input and subsequent changes were made based on the discussions between all authors. For example, articles that specifically discussed “organized” sports from the search strategy were selected for screening. Additionally, one article included a sample of adolescents aged 16 years and older with no upper bound. Articles that also included discussions within physical education/school settings were also selected for screening.

**Figure 1:**
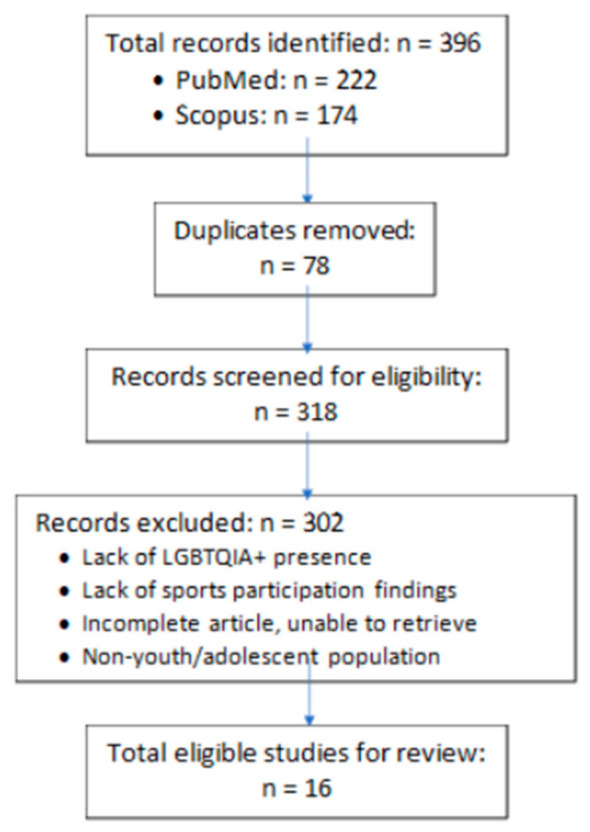
The literature search flow diagram.

Figure 1 details the study flow of the literature search and articles included for screening. A total of 396 records were identified, and 78 duplicate records were removed, leaving 318 records eligible for screening. A total of 16 articles were included in this final review.

## 3. Results

Sixteen articles were included in the final synthesis of this review. Table 1 provides a synthesis of key literature findings. Studies were categorized by sexual and gender identity groups to further highlight the current literature on specific groups.

### 3.1. Sexual and Gender Diverse Groups

One study surveyed LGB youth from the U.S., U.K., Canada, Australia, New Zealand, and Ireland (*n* = 1173) and explored the relationship between ‘coming out’ and experiences of homophobic behavior in team sports [4]. They found that almost half of the participants indicated being targeted by homophobic behavior, and those who ‘came out’ were significantly more likely to indicate being a target than those who did not [4]. Continuing the trend of increased exposure leading to increased risks for negative experiences, the study’s results indicated that a dose–response relationship of coming out to more people was associated with a greater chance of experiencing homophobic behavior [4].

A qualitative study conducted with LGBTQ+ youth aged 12 through 21 years of age from urban, rural, and coastal areas of England (*n* = 55) examined the lived experiences with physical activity insecurity [10]. Physical activity insecurity was found to be associated with larger public spaces, such as local gyms, sports clubs, and school environments. In addition to the feelings of insecurity within these spaces, participants expressed feelings of unsafety or discomfort, indicating that they felt more comfortable to be active in spaces that were simpler to navigate [10]. Intersecting barriers to engaging in physical activity were also identified through an inductive, reflexive thematic approach. Such barriers included deprivation, gender and sexuality, ethnicity, disability, accessibility, and affordability [10].

Data from a larger national sample of sexual and gender minority (SGM) youth (*n* = 9890) examined how physical activity interacts with bullying to influence depression and self-esteem [14]. Increased bullying was found to have higher depressive symptoms and lower self-esteem, and higher levels of physical activity were associated with lower depression and higher self-esteem. When the interaction between physical activity and bullying was examined, the findings were that physical activity did not mitigate the negative effects of bullying. The beneficial effects of physical activity were mainly seen in students that did not experience any bullying. Among youth that reported being bullied, their depression levels remained high regardless of their involvement in physical activity, along with small improvements in their self-esteem.

One study offered an international consensus statement that used available evidence on harassment and abuse in sports [15]. The consensus identifies psychological abuse as the most prevalent form of mistreatment in sport. Harassment and abuse occur across all sports and occur at disproportionately higher rates among child athletes, athletes with disabilities, and athletes that are a part of the LGBT+ community. The majority of perpetrators amongst children’s athletes are their peers. Evidence shows that the abuse is not explained by individual behaviors alone but is sustained by organizational cultures and coaches. They argue that effective abuse prevention begins at a systemic level with changes in leadership and culture changes [15].

Another study analyzed the relationship between participation and barriers in organized physical activity among students in urban Wisconsin (*n* = 4566) in grades 7–12 via a survey [16]. Using the results from the survey, students were categorized into four different barrier groups based on their reason for not participating in sports: high barrier, low barrier, bullied, and low interest. The high barrier class consisted of students with significant socioeconomic factors preventing them from playing sports. Of students who identified as LGBTQ+, 57% reported no participation in organized sports or group exercise, which was lower than that observed in cisgendered and heterosexual youth. Further analysis revealed that students who identified as bisexual, gay, lesbian, or pansexual all had an increased adjusted odds ratio of being in the bullied group when compared to students identified in the low barrier group. Additionally, students who identified as heterosexual had a 0.26 adjusted odds ratio of being in the bullied group [16].

### 3.2. Transgender and Gender Diverse Groups

One study that surveyed trans individuals in Australia aged 16 years or older (*n* = 664) explored bullying experiences, including barriers and facilitators, in sports and fitness [3]. They found that only one-third reported regularly participating in sports/fitness. Of these respondents, another third reported experiencing gender-based bullying [3]. Reported internal barriers included anxiety-related symptoms about others’ reactions to their identity, their own report of body dissatisfaction, and general fears of feeling accepted by others in the team. Participants reported reasons for not participating in sports are also due to inadequate bathrooms or changing facilities for transgender individuals, exclusionary regulations against transgender individuals, and invasive policies towards transgender individuals [3]. While many institutions and facilities are enhancing their inclusionary initiatives to affirm transgender individuals, several existing institutions and facilities have not yet made these initiatives. Such initiatives should be prioritized for sports federations, organizations, and policymakers.

Another study surveyed transgender girls in high schools across the United States (*n* = 294) and explored the reasons for participating and not participating in organized sports [13]. The survey was designed to explore reasons to participate and reasons not to participate in organized sports. Respondents indicated several reasons to participate in organized sports that align with the existing literature. These reasons included physical and mental health benefits; social connections and fun; family expectations; and, most interestingly, gender affirmation [13]. Reasons not to participate in organized sports were indicated as not being interested or athletic, having gendered teams and worsened gender dysphoria, physical or mental health limitations, social discomfort, bullying, and a lack of access due to gender identity [13]. There is a clear need for inclusive sports environments not just for all youth but for transgender youth in particular, given that some of the results of the study highlight bullying and worsened gender dysphoria.

One study that conduced a self-report school survey with transgender and gender diverse (TGD) adolescents in Minnesota in grades 9 through 11 (*n* = 10,454) examined the relationships of bias-based bullying due to sexual orientation, gender identity, and/or gender expression (SOGIE-BB) with depression and anxiety and sports participation [17]. In the first set of analyses, SOGIE-BB was found to be associated with elevated levels of self-reported depression and anxiety [17]. About half of respondents with reported depression and majority of respondents with reported anxiety also reported not participating in any organized sports [17]. It can then be concluded that those who experienced no SOGIE-BB were significantly associated with lower prevalences of depression- and anxiety-related symptoms. Additionally, sports participation was significantly associated with lower prevalences of depression- and anxiety-related symptoms [17]. Promotion of sports participation can not only promote overall well-being but can also help prevent SOGIE-BB experiences that are also associated with mental health risks.

Another study examined physical activity, team sports, and physical education between transgender and non-transgender European high school students (*n* = 156,369) and whether it was influenced by bullying [18]. Transgender youth were significantly less likely to engage in physical activity compared with non-transgender peers. However, participation in school-based physical education classes did not significantly differ between transgender and non-transgender youth. There was a higher prevalence of bullying between transgender youth and youth who were not sure of their gender modality. Despite this, after adjusting for bullying experiences, there was no change in relationship between transgender status and physical activity outcomes.

### 3.3. Gender Differences in Sports Participation

While the following studies do not focus on sexual and gender diverse (SGD) youth in particular, they provide insightful findings on gender differences in bullying experiences and sports participation. One study that examined the relationship between bullying and the type of physical activity practiced by pre-adolescents and adolescents aged 10 to 19 years in Spain (*n* = 2025) found that there is a higher rate of victimization in boys who did not practice physical activity [6]. Additionally, they found that there were higher levels of victimization and perpetration among boys who practiced wrestling compared to other physical activities. This may be related to the increase in contact and competitive nature of the activity. The overall findings of this study indicate that though participation in organized physical activities may be related to higher perpetration among adolescent boys, physical activity may also be a protective factor against bullying victimization [6].

One study that conducted a school-based survey with students in grades 9 through 12 in the U.S. (*n* = 20,103) examined the associations between witnessing violence and attending physical education (PE) classes or meeting PE guidelines [5]. They found positive associations between witnessing violence and meeting certain PE guidelines among male students, while there were negative associations between attending PE class and witnessing violence among female students [5]. Participation in physical activities and organized sports may serve as a motivating operation to help cope with such negative or violent experiences.

A study that consisted of 410 high school students from a martial arts middle school in China in early 2023 completed a follow-up survey in late 2023 [8]. The survey aimed to examine the relationship between sprint performance and bullying among adolescents while emphasizing the moderating role of sex. The results of the study revealed that longer, or slower, sprint times were significantly associated with higher levels of overall bullying victimization, peer ridicule, and physical altercations [8]. However, the researchers found a significant difference with sex, where boys with slower sprint times were faced with higher levels of bullying victimization, while there were no significant differences in bullying victimization based on sprint times for girls [8]. This provides more evidence on how physical fitness and sex interact to influence bullying victimization.

Another study that conducted a self-report survey in Italy with adolescents aged 13–21 years (*n* = 4829) examined the moderating relationship between sport participation and bullying as well as depressive symptoms [7]. Almost one quarter of respondents reported that they were bullied in the last year and yielded higher scores on a questionnaire that assessed depression-related symptoms [7]. These rates were more common among respondents who identified as female as well as those who do not engage in organized sports. The researchers conducted structural equation models to assess moderators (e.g., gender, exercise frequency, and sport participation) of the association between bullying and depression symptoms. They revealed that exercise frequency did not moderate the relationship between bullying and depression [7]. This speaks to the complexity of the presence of other social factors related to bullying and mental health despite the positive effects of physical activity. Participation in organized sports can serve as a buffer against the effects of bullying while also proving to be a strategy for increasing exercise and one’s mood.

One study analyzed coaches from various sports disciplines of athletes nationally aged 6–14 years (*n* = 30) by using a Q methodology to assess their perspectives on preventing peer bullying in sports [9]. The questionnaire revealed that the most positively ranked items in terms of reducing bullying were activities like sensitivity training and activities that increase social relationships. The most negatively ranked items included wording such as sensitivity training being unnecessary, open conversation with athletes to prevent bullying leads to negative outcomes, and rewarding positive behaviors to prevent bullying is not appropriate. Analysis revealed five strategy domains (education and awareness, discipline and sanctions, parental and social support, team dynamics and leadership, and observation with reward) that coaches should reinforce to help limit bullying in youth sports. Coaches describe education, mainly in the form of empathy development and sportsmanship training, as foundational in helping young athletes recognize the emotional impact of bullying before behaviors escalate.

A study that was conducted in Southern Spain analyzed the relationship between participation in sports and whether that will impact bullying victimization and aggression among adolescents (*n* = 1454) aged 12–16 years from secondary schools [12]. Students that initially experienced victimization were more likely to develop bullying habits later. However, students that participated in sports were less likely to display this pattern. This study suggests that participation in sports may function as a protective factor against developing bullying behaviors. Appearance-based stigmatization operates strongly within athletic and performance-oriented environments.

Finally, one study conducted a systematic review, which included 23 empirical studies, to identify factors associated with bullying behavior among children aged 5–17 years and adolescents in physical education settings [1]. The review identified six major categories that influence bullying behavior in physical education environments: demographic, physical movement, physical appearance, psycho-cognitive, teacher-related, and contextual factors. Gender differences fell under demographic factors, with male students being more likely than girls to perpetrate and experience bullying in physical education class [1]. Students whose body types deviated from perceived norms, such as students who were deemed too tall, short, overweight, or underweight, were more likely to experience bullying during physical education.

## 4. Discussion

As the existing literature highlights, bullying has been a public health concern for several decades, particularly among children and adolescents in school settings. It has longstanding negative effects on youths’ mental and emotional health, as well as their development and overall well-being, yet experiences look different across individuals according to the findings of studies. For example, some studies found no significant mitigating effects of physical activity on bullying or a moderating relationship between physical activity and bullying or depression yet indicated that sports participation can serve as a buffer [7,14]. These discordances in findings highlight that individual differences may be explained by a variety of factors, including study design, population/location, level of severity of bullying examined, or the conceptualization of bullying and organized sports participation. Nevertheless, schools are in a position to support and offer opportunities for students to be physically active throughout the school day to meet youth physical activity guidelines as proposed by the U.S. Centers for Disease Control and Prevention (CDC) [5]. Schools are equipped to address and prevent instances of bullying and to support youth who are experiencing bullying firsthand.

### 4.1. Clinical Implications

Understanding the current and negative experiences with physical activity among students with negative experiences will be useful for practitioners and administrators in influencing and creating infrastructures and programming within the schools toward safe, supportive, and inclusive environments for physical activity. Given the interactions between sex and athletic performance for specific types of bullying victimization, it is important that school environments develop anti-bullying strategies and emphasize the need for sex-specific interventions. Program development could include activities that enhance general strength, athleticism, and physical fitness to reduce the vulnerability to bullying [8]. Programs may also consider conflict resolution, self-esteem building, and fostering supportive peer relationships [8]. Additionally, administrators within the school system are encouraged to enforce policies or develop programs that aim at creating a safer space for LGB youth to participate in organized sports [4].

Prevention programs are designed as a means of prevention and treatment of bullying and victimization in the school setting. Such organized sports activities should aim to lower the risk of youths developing aggressive and defiant behaviors, and to improve the reduction in aggressiveness and the promotion of resilience from an anti-bullying approach [6]. Existing prevention programs consider PE within schools as a factor in the role against violence in school settings due to its valuable effects of social skills training and development [12]. As PE is an important contribution to healthy youth development, teachers serve a crucial role in the progress of their development. Teachers hold the position to help foster positive environments during PE courses, highlighting student empowerment and social empathy development [1]. Similarly, sports coaches should not only help students to improve their engagement and commitment to physical activity but also develop their social skills and growth in society. Additionally, local community sports organizations to develop programs to create safe spaces in which LGBTQ+ youth can feel welcomed to engage in physical activities together [10].

Coaches serve an important leadership role for student athletes. Coaches should model the leadership role by fostering mutual respect while aiming to reduce bullying behaviors. One strategy for encouraging leadership in students is rotating team captaincy to allow each student to experience leadership responsibilities [9]. Student athletes are then able to effectively develop essential problem-solving skills and skills to support and empower their teammates. Coaches also have a mentoring role in that they model these adaptive and leadership skills actively, gradually allowing the student athletes to take responsibility while guiding them along the way. Coach training programs that focus on conflict resolution and bullying intervention, as well as implementation of feedback mechanisms, should be integrated within schools to enhance anti-bullying strategies in sports [9]. Cultural competence training within the domain of working with LGBTQ+ individuals for coaches, administrators, and parents is recommended to promote this level of inclusivity [13]. Thus, understanding engagement or disengagement in physical activities can be useful for teachers, coaches, and health practitioners to further support youth [5]. The CDC provides resources for caregivers, students, and schools to learn about how to prevent, respond to, and gain skills to manage bullying [19].

### 4.2. Study Limitations and Directions for Future Research

While this review noted important considerations for understanding the relationship between bullying and sports participation among SGD youth, several limitations are also noted. The first limitation lies within the initial search strategy, involving both a preliminary and focused search that was then combined. This resulted in a number of articles that did not exclusively concern SGD youth but still contributed to important information surrounding the topic of bullying and sports participation. Additionally, the review relied on only two databases, limiting the breadth of the existing literature surrounding the topic and, thus, not enriching the search for the target population. The review also did not focus on quality appraisal of each study, and there is a lack of heterogeneity of designs.

The initial search goal on the topic of the relationship between bullying and sports participation among youth intended to also include youth of color (i.e., youth who identify as Black, Indigenous, People of Color) of all sexual and gender identities. Utilizing the search strategy detailed in Materials and Methods, studies that focused on BIPOC youth yielded little to no results. This is important to consider given the concept of intersectionality in which sexual and gender diverse youth may also identify as a youth of color. However, the intersectionality of BIPOC individuals of all sexual and gender identities yields varied differences in experiences with bullying and its impact on sports participation. The effects of bullying, as well as barriers and facilitators to participating in sports, may be more complex due to these multicultural identities and factors. To understand this dynamic, more research is needed in this area that is specific to the experiences of BIPOC youth related to bullying outcomes and sports participation.

Additionally, many of the articles included in the final review discuss gender differences in bullying and sports participation without examining differences among SGD youth. Thus, nearly half (about 44%) of the evidence in this narrative review speaks to the general youth population. This emphasizes the lack of literature around the topic of SGD youth, bullying, and sports participation. Further, one of the studies examined bullying and sports participation more broadly among an open upper age band. While there may be several attempts to engage relevant stakeholders—teachers, coaches, and parents—in enhancing anti-bullying strategies, there is a lack of evidence-based recommendations particularly for SGD youth. More research is required to understand the efficacy of such training programs and interventions to empower SGD youth athletes in organized sports.

## 5. Conclusions

Participation in organized sports can serve as both a facilitator and a barrier to improved mental health and overall well-being. Sexual and gender diverse (SGD) or transgender youth are much less likely than their peers to participate in organized sports due to several sociopsychological and systemic issues. Such issues include but are not limited to inadequate changing facilities, exclusionary regulations, invasive policies, and a lack of access. The presence of bullying, oftentimes in team sports, also plays a role in the influence of sports participation and engagement in physical activity for SGD youth. Such negative experiences are associated with increased symptoms of depression and anxiety, as well as decreased self-esteem. The findings from this narrative review are varied in experiences, highlighting the heterogeneous relationship between bullying and sports participation. However, many studies emphasize that schools and administrators should develop policies and programs to create safer spaces for SGD youth to feel welcomed in physical education courses and sports teams. Coaches, teachers, and health practitioners should stay informed on ways to augment these necessary changes to empower and encourage youth to engage in sports and physical activity. This is all possible with future research continuing to assess both the need for and effectiveness of inclusive sports programs.

## Figures and Tables

**Table 1:** Literature synthesis.

Author (Year)	Sample	Study Design	Relevant Findings	Conclusions
Sexual and Gender Diverse Youth				
Denison et al. (2021) [4]	LGB youth from the US, UK, Canada, Aus, NZ, and Ireland (*n* = 1173)	Online survey with logistic regression models	Overall, 41.6% have been targets of homophobic behavior. Participants who ‘came out’ to teammates were significantly more likely to report being targets of homophobic behavior. Dose–response relationship, with coming out to more people associated with greater likelihood of experiencing homophobic behavior.	LGB experiences of homophobic behavior appear common overall but are greater in those who have come out to teammates. Sports administrators and governments need to develop programs and enforce policies that create safe environments where LGB youth can participate in sports.
Dodd-Reynolds et al. (2024) [10]	LGBTQ+ youth aged 12–21 years from urban, rural, and coastal areas of England (*n* = 55)	Qualitative study with inductive, reflexive thematic approach	Felt more comfortable to be active in spaces that were simpler to navigate. Local gyms, sports clubs, and school environments often involved feelings of insecurity, unsafety, or discomfort. Intersecting barriers: deprivation, gender and sexuality, accessibility, disability, COVID-19, affordability, ethnicity, and proximity of social networks.	There is a need for safe spaces in which young people can come together within the local community and choose to be active.
Kirklewski et al. (2023) [14]	Youth athletes aged 11–17 years (*n* = 49,890)	Cross-sectional online survey	Physical activity levels are negatively related to depression and positively related to self-esteem. PA had no impact on depression and self-esteem in participants that were being bullied. For SGM youth who have been bullied, exercise has less of an effect on depression or self-esteem levels.	Future research should examine the interaction between overall school environment on bullying, self-esteem, and depression symptoms in SGM youth.
Mountjoy et al. (2016) [15]	Child athletes, athletes with disability, and LGB athletes	Consensus statement/guidelines	A systematic multiagency approach is most effective to reduce tolerance and prevent abuses. Reviews of law enforcement strategies; culturally tailored education for athletes, parents, athlete entourage, fans, sponsors, and sports administrators; and implementation of policy and procedures within the sports community are encouraged.	Suggestions for guidelines include appointing or working with qualified, designated personnel responsible for safe sport programming and athlete welfare; listening to the voices of athletes in decision-making about their own protection; and fostering strong partnerships with athletes’ parents/caregivers to promote safe sports.
Parchem et al. (2024) [16]	Urban Wisconsin LGTBTQ+ students grades 7–12 (*n* = 4566)	Cross-sectional study—secondary analysis of an existing dataset	When LGBTQ+ youth are not facing pervasive individual and systemic barriers, they may be more likely to participate in organized physical activity. A quarter of the sample reported experiencing bullying for their looks or LGBTQ+ status and not experiencing other barriers or disinterest.	Physical activity promotion among LGBTQ+ youth would be strengthened by policies that address inequitable access to opportunities and bias-based bullying. Free or reduced-fee physical activity opportunities sponsored by school districts or public programs are a clear intervention that would reduce access barriers.
Transgender and Gender Diverse Groups				
Bailey et al. (2024) [3]	Trans people aged ≥16 years living in Australia (*n* = 664)	Cross-sectional online survey	Overall, 32.8% participate in sports. Internal barriers: anxiety about others’ reactions, body dissatisfaction, and fears about feeling accepted. Inadequate bathroom/changing facilities, exclusionary regulations, and invasive policies. A total of 34.4% experienced gender-based bullying.	Trans people face barriers to sports participation despite mental health benefits to participation. Affirming trans people in sports should be prioritized by sports federations, organizations, and policymakers.
DeChants et al. (2024) [13]	Transgender girls in high school (*n* = 294)	Descriptive, exploratory survey	Reasons to participate: physical health benefits, mental health benefits, fun, social connections, family expectations, and gender affirmation. Reasons not to participate: not interested, gendered teams, not athletic, physical or mental health limitations, social discomfort, worsened gender dysphoria, bullying, and lack of access.	There is a need for inclusive sports environments for transgender athletes. Providing LGBTQ+ cultural competence training for coaches, administrators, and parents may decrease barriers and increase comfort.
Kaja et al. (2025) [17]	Transgender and gender diverse (TGD) adolescents in 9th, 10th, and 11th grades (*n* = 10,454)	Self-report school survey	Bias-based bullying due to sexual orientation, gender identity, and/or gender expression (SOGIE-BB) associated with elevated depression and anxiety. Seven groups with depression and three out of four groups with anxiety reported no sports participation. Experiencing no SOGIE-BB was significantly associated with lower prevalences of elevated depression and anxiety symptoms. Sports participation was significantly associated with lower prevalences of elevated depression and anxiety symptoms.	Experiencing SOGIE-BB is associated with higher mental health risks, while sports participation is associated with better mental health among TGD adolescents. Promoting sports participation and preventing SOGIE-BB could enhance TGD adolescents’ overall well-being.
Voss et al. (2023) [18]	European secondary school students grades 9–12 (*n* = 156,369)	Cross-sectional survey via self-report questionnaire	Higher prevalence of bullying among transgender youth and youth who were not sure of their gender modality. Transgender youth reported similar odds of PE participation as non-transgender youth.	Further investigation into transgender youths’ satisfaction and comfort in PE settings, as well as their engagement in PE, would help ensure these youth have positive and meaningful PE experiences.
Gender Differences in Sports Participation				
Zhou et al. (2023) [1]	A total of 23 articles	Systematic review	Increased sedentary behaviors seem to hinder the development of interpersonal relationships, pro-social behavior, and conflict resolution abilities making them more susceptible to bullying. Boys who embodied masculinities with characteristics that did not match the requirements of the social setting were marginalized.	The influence of physical appearance emerged as a significant contributor to bullying behaviors, with body shape- and appearance-related factors playing a substantial role in victimization experiences. Physical movement factors, such as physical activity, sedentary behavior, and sports competence, were found to be associated with various forms of victimization.
Cornett et al. (2024) [5]	Students in grades 9–12 in the U.S. (*n* = 20,103)	Cross-sectional, school-based survey conducted biennially since 1991	Positive association between witnessing violence and meeting aerobic and muscle-strengthening guidelines among males. Negative association between attending PE and witnessing violence among females. Negative association between attending PE and being bullied among males.	PA might serve as a mechanism that students employ to cope with negative safety and violent experiences. Understanding PA behaviors will be useful for school leaders, teachers, and public health practitioners.
Benitez-Sillero et al. (2023) [6]	Students aged 10–19 years in Spain (*n* = 2025)	Descriptive, exploratory, cross-sectional study with non-probabilistic sampling	Higher rate of victimization in boys who did not practice PA.	PA may be a protective factor against bullying victimization, especially in boys. However, participation in organized sports may be related to higher perpetration.
Holbrook et al. (2020) [7]	Youth aged 13–21 years in Italy (*n* = 4829)	Self-report Epidemiologia dell-Infortunistica Stradale (EDIT) survey	Overall, 18.7% were bullied in the past year and scored higher in depression. Bullying and depression were more related in females and those not playing sports. Exercise frequency did not moderate the relation between bullying and depression.	Sports participation buffers against the effects of bullying and may prove a helpful strategy for increasing exercise, positive peer interactions, and mood in adolescents.
Han et al. (2025) [8]	High school students who attended martial arts middle school in China (*n* = 410)	Two-wave follow-up survey	Longer sprints associated with higher levels of bullying victimization. Boys with slower sprints faced higher levels of bullying victimization. No significant differences in bullying victimization based on sprint times for girls.	There is a need for sex-specific anti-bullying interventions in school environments. Understanding and addressing the unique factors that influence bullying among adolescents can help schools develop effective strategies to reduce victimization and promote safer environments.
Karafil & Pehlivan (2025) [9]	Coaches from various sports disciplines of athletes aged 6–14 years (*n* = 30)	Q methodology that integrates both qualitative and quantitative analysis (both surveys and standardized questionnaires)	Coaches employ five key domains to manage peer bullying: education and awareness (sportsmanship training), discipline and sanctions, parental and social support, team dynamics and leadership, and observation and reinforcement. More coach involvement with parents led to decreased bullying.	Coaches play a multifaceted role in bullying prevention, and a holistic approach that integrates education, prevention, and structured discipline is key to limiting bullying.
Ortiz-Marcos et al. (2022) [12]	Adolescents aged 12–16 years from Southern Spain (*n* = 1454)	Cross-sectional survey via self-report questionnaire	Victim behaviors are not associated with aggressor behaviors when practicing sport. Victim behaviors are associated with future aggressor behaviors when not practicing sport. The practice of sport promotes responsibility and improves coexistence among the peer group.	Schools should implement sports intervention programs for the purpose of eliminating bullying in the classroom. Sports can help eliminate “the spiral of violence.”

## Data Availability

No new data were created or analyzed in this study. Data sharing is not applicable to this article.

## Abbreviations

The following abbreviations are used in this manuscript:

LGBTQIA+	Lesbian, Gay, Bisexual, Transgender, Queer, Intersex, Asexual
WHO	World Health Organization
BIPOC	Black, Indigenous, People of Color
CDC	Center for Disease Control and Prevention
LBG	Lesbian, Bisexual, Gay
SOGIE-BB	Sexual Orientation, Gender Identity, and Expression—Biased-Based Bullying
SGD	Sexual and Gender Diverse
PE	Physical Education

## Appendix A. Literature Search Strategies

The following searches were conducted in PubMed on 1.14.26 and filtered to the last 10 years:

Preliminary search—bullying and youth sports

(“Bullying”[Mesh] OR bully* OR bullie*) AND (“Adolescent”[Mesh] OR adolescent* OR teen* OR youth*) AND (“Sports”[Mesh] OR “Youth Sports”[Mesh] OR sport* OR “youth sport*”)

- A total of 222 results

Focused search—bullying and youth sports, LGBTQ+ youth/youth of color

(“Bullying”[Mesh] OR bully* OR bullie*) AND (“Adolescent”[Mesh] OR adolescent* OR teen* OR youth*) AND (“Sports”[Mesh] OR “Youth Sports”[Mesh] OR sport* OR “youth sport*”) AND (“Sexual and Gender Minorities”[Mesh] OR “Ethnic and Racial Minorities”[Mesh] OR LGBTQIA OR LGBTQ OR gay OR lesbian OR bisexual OR transgender OR queer OR intersex OR asexual OR “gender minorit*” OR “sexual minorit*” OR minorit* OR race OR racial OR ethnic* OR “person of color” OR “people of color” OR “youth of color” OR BIPOC OR marginalized OR “marginalized communit*”)

- A total of 33 results

Combined searches

((“Bullying”[Mesh] OR bully* OR bullie*) AND (“Adolescent”[Mesh] OR adolescent* OR teen* OR youth*) AND (“Sports”[Mesh] OR “Youth Sports”[Mesh] OR sport* OR “youth sport*”)) OR ((“Bullying”[Mesh] OR bully* OR bullie*) AND (“Adolescent”[Mesh] OR adolescent* OR teen* OR youth*) AND (“Sports”[Mesh] OR “Youth Sports”[Mesh] OR sport* OR “youth sport*”) AND (“Sexual and Gender Minorities”[Mesh] OR “Ethnic and Racial Minorities”[Mesh] OR LGBTQIA OR LGBTQ OR gay OR lesbian OR bisexual OR transgender OR queer OR intersex OR asexual OR “gender minorit*” OR “sexual minorit*” OR minorit* OR race OR racial OR ethnic* OR “person of color” OR “people of color” OR “youth of color” OR BIPOC OR marginalized OR “marginalized communit*”))

- A total of 222 results

The following searches were conducted in Scopus on 1.14.26 and filtered to the last 10 years:

Preliminary search—bullying and youth sports—title/abstract/keyword

(bully* OR bullie*) AND (adolescent* OR teen* OR youth*) AND (sport* OR “youth sport*”)

- A total of 174 results

Focused search—bullying and youth sports, LGBTQ+ youth/youth of color

(bully* OR bullie*) AND (adolescent* OR teen* OR youth*) AND (sport* OR “youth sport*”) AND (LGBTQIA OR LGBTQ OR gay OR lesbian OR bisexual OR transgender OR queer OR intersex OR asexual OR “gender minorit*” OR “sexual minorit*” OR minorit* OR race OR racial OR ethnic* OR “person of color” OR “people of color” OR “youth of color” OR BIPOC OR marginalized OR “marginalized communit*”)

- A total of 30 results

Combined searches

((bully* OR bullie*) AND (adolescent* OR teen* OR youth*) AND (sport* OR “youth sport*”)) OR ((bully* OR bullie*) AND (adolescent* OR teen* OR youth*) AND (sport* OR “youth sport*”) AND (LGBTQIA OR LGBTQ OR gay OR lesbian OR bisexual OR transgender OR queer OR intersex OR asexual OR “gender minorit*” OR “sexual minorit*” OR minorit* OR race OR racial OR ethnic* OR “person of color” OR “people of color” OR “youth of color” OR BIPOC OR marginalized OR “marginalized communit*”))

- A total of 174 results
